# Endoskopieren in der Schwangerschaft (EndiS)

**DOI:** 10.1055/a-2677-1877

**Published:** 2025-11-10

**Authors:** Henrike Lenzen, Lukas Welsch, Stefanie Reichermeier, Jutta Keller

**Affiliations:** 1 9177 Klinik für Gastroenterologie, Hepatologie, Infektiologie und Endokrinologie, Medizinische Hochschule Hannover, Hannover, Germany; 2 39726 Klinik für Gastroenterologie, Hepatologie, Interventionelle Endoskopie und Diabetologie, Städtisches Klinikum Braunschweig gGmbH, Braunschweig, Germany; 3 39790 Klinik für Gastroenterologie, Diabetologie und Infektiologie, Klinikum Hanau gGmbH, Hanau, Germany; 4 9146 Medizinische Klinik II, Gastroenterologie, Onkologie, Endokrinologie und Infektiologie, Klinikum St Marien Amberg, Amberg, Germany; 5 Medizinische Klinik, Israelitisches Krankenhaus, Hamburg, Germany

**Keywords:** Endoskopie, Karriere, Mutterschutzgesetz, Elternzeit, Weiterbildung, Endoskopikerin, Gastroenterologie, Schwangerschaft, endoscopy, career, female physician, maternity protection act, maternity leave, pregnancy, endoscopist, gastroenterology

## Abstract

Die demografische Struktur der Ärzteschaft* ist im Wandel. Bedingt durch einen stetigen Zuwachs an weiblichen Studierenden ist auch die Zahl der Ärztinnen, die eine gastroenterologische Facharztausbildung beginnen, deutlich gestiegen. Viele von ihnen treten während ihrer gebärfähigen Jahre in das Fachgebiet ein. Gleichzeitig stellt die Endoskopie eine zentrale Rolle im beruflichen Alltag von Gastroenterologinnen dar. Allerdings führt noch viel zu häufig die Annahme einer Familienplanung oder die Bekanntgabe der Schwangerschaft zu Beeinträchtigungen der Weiterbildung und des Karriereweges 1 2 , insbesondere auch im Hinblick auf den Endoskopie-Einsatz. Dies führt oft zu einer späten Kommunikation der Schwangerschaft, teilweise wird die Familienplanung auch auf einen späteren Zeitpunkt bis nach der Facharztausbildung verschoben 3 . Es besteht daher ein wachsender Bedarf, sich mit berufsbezogenen Herausforderungen, wie der Durchführung endoskopischer Verfahren während der Schwangerschaft und Stillzeit, auseinanderzusetzen. Dieses Statement, ergänzt durch ein Rechtsgutachten sowie praxisnahe Checklisten und weiterführende Informationen auf der DGVS Webseite†, sollen mehr Klarheit und Orientierungshilfe bieten, um die Sicherheit und das Wohlergehen schwangerer und stillender Ärztinnen zu gewährleisten und gleichzeitig hohe Standards in der Patientenversorgung zu wahren. Ziel ist es, Karrierechancen für die schwangeren Ärztinnen zu sichern und ein gleichberechtigtes Entwicklungspotential, auch im Sinne der zukünftigen Patientenversorgung, zu fördern.

## Abstract (en)

The demographic structure of the medical profession* is changing. Due to a steady increase in female students, the proportion of female gastroenterologists and the number of female doctors starting specialist training in gastroenterology has increased significantly. Most of these women enter the speciality training in their childbearing years. At the same time, endoscopy plays a central role in the daily work of gastroenterologists. However, far too often the assumption of family planning or the announcement of pregnancy still leads to impairments in further training and career paths 1 2 , particularly with regard to the endoscopy training. This still all too often results in delayed communication of pregnancy and in some cases, family planning is postponed until after the specialist training 3 . Therefore, there is a growing need to address specific professional challenges such as performing endoscopic procedures during pregnancy and breastfeeding. This statement, a legal opinion, practical checklists and further information on the DGVS website† aim to provide more clarity and guidance on this issue, ensuring the safety and wellbeing of pregnant and breastfeeding doctors while maintaining high patient care standards. The aim is to ensure career opportunities for pregnant doctors and to promote equal development potential, also in terms of future patient care.

## Aktuelle Situation und rechtliche Grundlagen zum Endoskopieren in der Schwangerschaft

Die demografische Entwicklung der Ärzteschaft* zeigt einen kontinuierlichen Anstieg des Frauenanteils unter den Studierenden und Ärzten, insbesondere auch in der Gastroenterologie. Viele Ärztinnen beginnen die Weiterbildung während ihrer gebärfähigen Jahre, ein Umstand, der mit spezifischen Herausforderungen verbunden ist. Gleichzeitig spielt die Endoskopie eine zentrale Rolle im beruflichen Alltag von Gastroenterologen. Die Annahme einer Familienplanung oder die Bekanntgabe einer Schwangerschaft führen allerdings noch viel zu häufig zu Beeinträchtigungen der Weiterbildung und des Karriereweges 1 2 , insbesondere auch im Hinblick auf den Einsatz in der Endoskopie. Ärztinnen berichten daher von einer verspäteten Mitteilung ihrer Schwangerschaft oder einer bewussten Verschiebung der Familienplanung bis nach der Facharztausbildung 3 . Dies unterstreicht den Bedarf nach klaren Regelungen und praxisnaher Orientierung, um die berufliche Entwicklung und die Karrierechancen für die schwangeren Ärztinnen zu sichern und ein gleichberechtigtes Entwicklungspotential, auch im Sinne der zukünftigen Patientenversorgung, zu fördern.

Das Mutterschutzgesetz (MuSchG) schützt die Gesundheit der berufstätigen werdenden und stillenden Mütter am Arbeitsplatz und in der Ausbildung. Gleichzeitig soll es die berufliche Sicherheit von schwangeren und stillenden Frauen gewährleisten. Die Regelungen des MuSchG werden ergänzt durch die Verordnung zum Schutze der Mütter am Arbeitsplatz (MuSchArbV). Dies sind im Speziellen Beschäftigungsverbot, Arbeitszeiten, Gesundheitsschutz, Kündigungsschutz, Mutterschaftsgeld und Stillzeiten. Die Überwachung und Einhaltung von MuSchG und MuSchArbV obliegt den nach dem Landesrecht zuständigen Aufsichtsbehörden, den staatlichen Ämtern für Arbeitsschutz. Mit der Novelle wurden die Arbeitgeber verpflichtet, eine Gefährdungsbeurteilung durchzuführen, um den Arbeitsplatz bzw. die Arbeitsbedingungen umzustrukturieren und so Beschäftigungsverbote zu vermeiden. Beschäftigungsverbote sind künftig auch nicht mehr einfach gegen den Willen der schwangeren Frauen möglich. Stattdessen sollen Arbeitsplätze umgestaltet werden, um Gefährdungen für die Schwangere und das ungeborene Kind auszuschließen. § 6 Abs. 1 MuSchG erlaubt auch die Beschäftigung an Sonn- und Feiertagen auf freiwilliger Basis, wenn bestimmte Voraussetzungen erfüllt sind. Seit der Novellierung des MuSchG im Jahr 2018 sind auch Studierende und Auszubildende in das Gesetz einbezogen, ausgenommen sind weiterhin Selbstständige. Bei selbstständig tätigen Ärztinnen fehlen gesetzliche Schutzmechanismen wie sie im Mutterschutzgesetz für Angestellte vorgesehen sind. Dies betrifft nicht nur die Gefährdungsbeurteilung und arbeitsmedizinische Betreuung, sondern auch organisatorische Aspekte wie Vertretungslösungen oder sozialversicherungsrechtliche Absicherung im Fall eines Tätigkeitsausfalls. Die berufliche Vereinbarkeit von Schwangerschaft und medizinischer Tätigkeit stellt für diese Gruppe daher eine besonders hohe Hürde dar.

Weitere wichtige und zu beachtende allgemeine Schutzvorschriften im Hinblick auf besondere Tätigkeiten in der Endoskopie sind u.a. in der Biostoffverordnung (BioStoffV), der Gefahrstoffverordnung (GefStoffV) und der Strahlenschutzverordnung (StrlSchV) geregelt. Wir verweisen hier auf die detaillierte Beschreibung im Rechtsgutachten, mit speziellem Schwerpunkt auf das Endoskopieren in der Schwangerschaft in der Gastroenterologie ^#^ . Das 2018 novellierte MuSchG führt allerdings nicht selten zu grundlegenden Einschränkungen und Verunsicherungen für Ärztinnen und Studentinnen, die dadurch in ihrem beruflichen Karriereweg behindert werden. Eine aktuelle Studie einer Online-Befragung konnte zeigen, dass etwa die Hälfte der befragten Ärztinnen Bedenken hatte, ihre Schwangerschaft dem Arbeitgeber zu melden. Gründe dafür sind vor allem die Sorge, Einschränkungen auf dem weiteren Karriereweg und bei der Weiterbildung zur Fachärztin hinnehmen zu müssen, ein Verbot von Operationen oder sonstige Tätigkeitsverbote. Sobald die Ärztinnen ihre Schwangerschaft meldeten, arbeiteten zwei Drittel der Ärztinnen maximal 50% ihrer bisherigen beruflichen Tätigkeit, wobei 18% der Ärztinnen den Sinn dieser Einschränkungen dabei nicht nachvollziehen konnten 4 .

In den letzten Jahren haben verschiedene Verbände und wissenschaftliche Vereinigungen bereits Positionspapiere und Leitfäden zur Verbesserung der Lage von schwangeren Ärztinnen entwickelt. Das Junge Forum O und U (JF-OU) der Deutschen Gesellschaft für Orthopädie und Unfallchirurgie (DGOU) hat die Initiative „Operieren in der Schwangerschaft“ (OPidS) gegründet, um die Arbeitsbedingungen und die Vereinbarkeit von Familie und Beruf für Chirurginnen in der Schwangerschaft in Deutschland zu verbessern ( www.opids.de ; Gründerinnen Dr. med. Maya Niethard und Dr. med. Stefanie Donner). Die Arbeitsgruppe „Arbeiten in der Schwangerschaft auf der Intensivstation“ der Deutschen Interdisziplinären Vereinigung für Intensiv- und Notfallmedizin (DIVI e. V.) hat 2024 ebenfalls ein Positionspapier mit Empfehlungen zur Verbesserung der Lage von schwangeren Mitarbeiterinnen auf einer Intensivstation veröffentlicht 5 . Der Deutsche Ärztinnenbund e. V. (DÄB) unterstützt und fördert seit Jahren Aktivitäten zur Verbesserung der Arbeitsbedingungen von Ärztinnen und Studentinnen in der Schwangerschaft und stellt Hinweise und Empfehlungen zu diesem Themenkomplex bereit ( www.aerztinnenbund.de ).

## Schwangerschaftsmeldung

In Deutschland besteht keine gesetzliche Pflicht für Schwangere, ihre Schwangerschaft dem Arbeitgeber mitzuteilen, allerdings wird eine frühzeitige Mitteilung empfohlen, um den gesetzlichen Schutz nach dem MuSchG zu gewährleisten. Nach Bekanntgabe der Schwangerschaft muss der Arbeitgeber die Schwangerschaft und den voraussichtlichen Entbindungstermin umgehend der Aufsichtsbehörde melden und gegebenenfalls Details zur Art der Beschäftigung und zu den Arbeitsbedingungen bereitstellen. Zudem ist der Arbeitgeber nach § 10 MuSchG verpflichtet, eine individuelle Gefährdungsbeurteilung des Arbeitsplatzes durchzuführen und entsprechende Schutzmaßnahmen zu ergreifen.

## Gefährdungsbeurteilung

Damit schwangere und stillende Ärztinnen ihre Weiterbildung oder Beschäftigung fortsetzen können, ist der Arbeitgeber nach § 10 Abs. 1 Nr. 1 MuSchG (sinnvoll in Verbindung mit der vorgeschriebenen Gefährdungsbeurteilung nach § 5 Arbeitsschutzgesetz (ArbSchG)) verpflichtet, eine anlassunabhängige Beurteilung der Arbeitsbedingungen durchzuführen und zu ermitteln, ob in einem Arbeitsbereich Gefährdungen für eine schwangere oder stillende Frau vorliegen können. Dies ist unabhängig von einer konkreten Schwangerschaft oder der Beschäftigung einer schwangeren oder stillenden Frau. Dabei wird ermittelt, ob bei einer Tätigkeit oder in einem Arbeitsbereich Gefährdungen für schwangere Ärztinnen auftreten können. Die Gefährdungsbeurteilung ist tätigkeitsspezifisch für die verschiedenen Arbeitsbereiche von Ärztinnen wie Endoskopie, OP, Station, Ambulanz etc. durchzuführen. Entsprechende Arbeitshilfen und Ratgeber stellt die DGVS auf ihrer Webseite zur Verfügung ^†^ . Bei der Gefährdungsbeurteilung nach § 5 ArbSchG muss der Arbeitgeber alle Arbeitsplätze auch bezüglich einer aerogenen Infektionsgefährdung durch SARS-CoV-2 beurteilen und Schutzmaßnahmen für alle Beschäftigten festlegen. Der Ausschuss für Mutterschutz (AfMu) beim Bundesministerium für Familie, Senioren, Frauen und Jugend (BMFSFJ) hat die „Empfehlung zur mutterschutzrechtlichen Bewertung von Gefährdungen durch SARS-CoV-2“ hierzu veröffentlicht 6 . Die Verletzung der Pflicht des Arbeitgebers zur Durchführung einer Gefährdungsbeurteilung kann als Ordnungswidrigkeit geahndet werden.

Nach Bekanntgabe der Schwangerschaft ist der Arbeitgeber nach § 10 MuSchG verpflichtet, zusätzlich eine individuelle Gefährdungsbeurteilung des Arbeitsplatzes durchzuführen.

## Schutzmaßnahmen

Basierend auf der Gefährdungsbeurteilung sind geeignete Schutzmaßnahmen festzulegen (technisch, organisatorisch, persönlich) und zu dokumentieren (§ 14 MuSchG). Dies erfolgt oft in Zusammenarbeit mit dem Betriebsarzt und ermöglicht die Beschäftigung einer schwangeren und stillenden Ärztin mit der entsprechenden Tätigkeit. Dies ist sowohl anlassunabhängig (Stufe 1) als auch anlassbezogen (Stufe 2) durchzuführen. Die anlassbezogene Gefährdungsbeurteilung muss für jede Schwangere und Stillende durchgeführt werden. Die Maßnahmen sind konkret für eine Schwangere festzulegen, Art und Umfang der Schutzmaßnahmen ergeben sich aus der Gefährdungsbeurteilung. Diese Maßnahmen werden dem zuständigen Gewerbeaufsichtsamt übermittelt, das deren Angemessenheit prüft und gegebenenfalls auch bei Nichteinhaltung der Maßnahmen für die gesetzlichen Schutzvorschriften ein Beschäftigungsverbot aussprechen kann.

Insbesondere bei einem Einsatz in der Endoskopie lassen sich Schutzmaßnahmen für die schwangere Ärztin sehr gut planen und umsetzen. Es handelt sich bei den Endoskopie-Untersuchungen in den meisten Fällen um immer wiederkehrende, bekannte Eingriffe und Prozeduren, bei denen einheitliche Konzepte und Schulungen zum Gebrauch der persönlichen Schutzausrüstung (PSA) etabliert sind. Endoskopische Notfalluntersuchungen, Endoskopien bei infektiösen Patienten und auch der Umgang mit und die Anwendung von spitzen Gegenständen sind überschaubar und können daher gut vermieden werden, im Gegensatz zu vielen anderen Arbeitsplätzen in der Gastroenterologie.

## Beschäftigungsverbot

Der Arbeitgeber hat die Arbeitsbedingungen so zu gestalten, dass eine Gefährdung einer schwangeren oder stillenden Frau oder ihres Kindes so weit wie möglich vermieden wird. Eine unverantwortbare Gefährdung muss ausgeschlossen sein. Sie gilt dabei als ausgeschlossen, wenn der Arbeitgeber alle Vorschriften einhält, die aller Wahrscheinlichkeit nach dazu führen, dass die Gesundheit einer schwangeren oder stillenden Frau oder ihres Kindes nicht beeinträchtigt wird. Sollte eine unverantwortbare Gefährdung ermittelt werden, so gilt es zuerst, geeignete Schutzmaßnahmen zu ermitteln und zu prüfen, ob der Arbeitsplatz angepasst oder umgestaltet werden kann. Erst wenn eine Anpassung nicht möglich oder unzumutbar ist, muss ein Arbeitsplatzwechsel erfolgen oder, falls dies auch nicht umsetzbar ist, ein teilweises oder vollständiges betriebliches Beschäftigungsverbot ausgesprochen werden.

Werdende Mütter dürfen nicht beschäftigt werden, wenn laut ärztlichem Zeugnis das Leben oder die Gesundheit von Mutter und Kind durch die Fortführung der Beschäftigung gefährdet ist (§ 16 des MuSchG, ärztliches Beschäftigungsverbot). Zudem gilt ein generelles Beschäftigungsverbot in den letzten sechs Wochen vor der Entbindung, es sei denn, die Schwangere erklärt sich ausdrücklich zur Arbeitsleistung bereit, wobei diese Erklärung jederzeit widerrufbar ist. Ergänzt werden diese Bestimmungen durch § 11 MuSchG, der werdende Mütter im Bereich vor bestimmten Tätigkeiten schützt, wie vor schweren körperlichen Arbeiten und dem Umgang mit gesundheitsgefährdenden Stoffen, Strahlen und Gasen. Auch ständige Steharbeit über vier Stunden nach dem fünften Schwangerschaftsmonat sowie Arbeiten, die ein erhöhtes Risiko für Berufskrankheiten darstellen, sind verboten. Schwangere und stillende Mütter dürfen keinen unverantwortbaren Gefährdungen durch Biostoffe der Risikogruppen 3 und 4 ausgesetzt werden (BioStoffV). Bei der Gefährdungsbeurteilung nach § 5 ArbSchG muss der Arbeitgeber auch alle Arbeitsplätze bezüglich einer aerogenen Infektionsgefährdung durch SARS-CoV-2 beurteilen und Schutzmaßnahmen für alle Beschäftigten festlegen.

Ein Beschäftigungsverbot in der Schwangerschaft oder in der Stillzeit ist somit selten notwendig. Eine Weiterbeschäftigung, insbesondere auch im Arbeitsbereich der Endoskopie, ist daher sehr gut möglich, es bedarf nur der Einhaltung genannter Maßnahmen und entsprechender Schutzmaßnahmen und in besonderer Weise einer individuellen Unterstützung durch den Arbeitgeber und das Team. In Bezug auf eine fortgesetzte operative Tätigkeit einer schwangeren Arbeitnehmerin oder Ärztin gibt es in der aktuellen Rechtsprechung bisher keinen bekannten Fall, in dem ein Arbeitgeber für Schäden verantwortlich gemacht wurde, die während dieser operativen Tätigkeiten entstanden sind. Die Zeit des Beschäftigungsverbots gilt nicht als Weiterbildungszeit.

## Haftung im Schadensfall

Der Arbeitgeber ist verpflichtet, die in der individuellen Gefährdungsbeurteilung festgelegten Schutzmaßnahmen bereitzustellen und deren Einhaltung zu überwachen oder die Überwachungsaufgaben zu delegieren. Diese Maßnahme ermöglicht dem Arbeitgeber, sich von der Haftung zu entlasten, falls Dritte, wie beispielsweise Patienten, Ansprüche geltend machen. Die schwangere Arbeitnehmerin trägt die persönliche Verantwortung für die Befolgung dieser Schutzmaßnahmen. Bei Verstößen des Arbeitgebers gegen das MuSchG können Strafzahlungen und im Falle vorsätzlicher Verstöße sogar härtere Strafen gemäß § 32 MuSchG drohen.

## Einsatz von schwangeren Ärztinnen in der Endoskopie

Der Einsatz in der Endoskopie basiert immer auf der freiwilligen Entscheidung der einzelnen Ärztin und es muss eine medizinische Unbedenklichkeit gegenüber dem Einsatz in der Endoskopie herrschen (Gynäkologie, Betriebsärztlicher Dienst). Eine individuelle Gefährdungsbeurteilung ihrer endoskopischen Tätigkeit muss erfolgen ^†^ . In enger Zusammenarbeit und Absprache mit der schwangeren oder stillenden Ärztin selbst, dem Arbeitgeber und dem zuständigen Betriebsarzt vor Ort soll so ein Einsatz in der Endoskopie unter sicheren Bedingungen ermöglicht werden. Dadurch ist eine erhöhte Transparenz in Bezug auf die Bekanntgabe der Schwangerschaft und somit eine Vermeidung von Mehrbelastung in der Frühschwangerschaft zu erwarten. Gemäß dem MuSchG sind die Rechte von schwangeren Frauen am Arbeitsplatz klar definiert, was auch für Ärztinnen in der Gastroenterologie gilt. Es müssen individuelle Gefährdungsbeurteilungen durchgeführt werden, und bestimmte Arbeitsbedingungen, wie das Vermeiden von Nacht- und Notfalldiensten, sind einzuhalten. Jede Tätigkeit einer schwangeren Ärztin in der Endoskopie basiert auf Freiwilligkeit und muss medizinisch unbedenklich sein. Die schwangere Ärztin kann ihren erklärten Wunsch, weiter endoskopieren zu dürfen, jederzeit mit sofortiger Wirkung zurücknehmen.

Bisherige Studien, die die Schwangerschaftsrisiken bei Ärztinnen untersuchten, konzentrierten sich in erster Linie auf Schwangerschaftskomplikationen wie Präeklampsie, vorzeitige Wehen und Schwangerschaftsfolgen wie Fehlgeburten, Totgeburten und Kaiserschnittraten 7 8 . Allerdings fehlen prospektive, risikoangepasste Beobachtungsstudien über die Schwangerschaft von Ärztinnen. Nur wenige Studien haben sich mit den spezifischen Auswirkungen der Berufsausübung von Ärztinnen beschäftigt, und keine hat sich speziell mit der Durchführung von Endoskopien durch schwangere Gastroenterologinnen befasst. Bisher unveröffentlichte Daten aus der JUGA Study Group legen keine Zunahme an Schwangerschaftskomplikationen durch Endoskopieren in der Schwangerschaft nahe.

Mit Bekanntgabe der Schwangerschaft treten jedoch nicht selten für die schwangere Ärztin Probleme und Herausforderungen bezüglich des Einsatzes in der Endoskopie auf. Die Rotation in die Endoskopie wird aufgrund möglicher Arbeitszeitverkürzungen und möglicher antizipierter geringerer Flexibilität weiter verschoben und findet in der Regel auch nicht direkt nach der Schwangerschaft statt. Eine kontinuierliche Weiterbeschäftigung unterstützt jedoch nicht nur die Weiterbildung und Karriere der schwangeren oder stillenden Ärztin, sondern führt auch zu einer besseren Personalbindung, einer Kontinuität im Personalpool und damit zu einem wichtigen Qualitäts- und Kompetenzerhalt in der Patientenversorgung und zu einem Mehrwert für alle Beteiligten. Insgesamt ist es daher zwingend notwendig, dass nicht nur erst bei Bekanntmachung der Schwangerschaft ein Gespräch seitens des Arbeitgebers und der Vorgesetzten angeboten und geführt wird, sondern bereits frühzeitig in der Beschäftigung der Ärztin allgemeine und betriebsinterne Regelungen kommuniziert werden und Weiterbildungsinhalte, Rotationen und Karriereentwicklung speziell auch im Rahmen einer Familienplanung besprochen werden.

Zur Unterstützung von Ärztinnen in der Schwangerschaft können innerklinische Mentoring-Programme sowie psychosoziale Beratungsangebote beitragen, die einen geschützten Raum für den Austausch über individuelle Belastungen und berufliche Perspektiven schaffen. Ergänzend stellen Supervisionen, interdisziplinäre Unterstützungsteams und niedrigschwellige Gesprächsangebote durch Betriebsmedizin, Abteilungsleitung oder externe Fachstellen wichtige Ressourcen sowie strukturierte Rückkehrgespräche nach Schwangerschaft und Elternzeit dar. Eine frühzeitige Integration solcher Maßnahmen kann wesentlich zur psychischen Stabilität und zur Aufrechterhaltung beruflicher Kontinuität beitragen.

## Überlegungen zur Durchführung der ERCP in der Schwangerschaft

Die endoskopische retrograde Cholangiopankreatografie (ERCP) und allgemein durchleuchtungsgestützte endoskopische Untersuchungen bergen bestimmte zusätzliche Risiken für die durchführende Endoskopikerin in der Schwangerschaft. Die Risiken einer Strahlenexposition für den sich entwickelnden Fötus sind hinlänglich bekannt und umfassen Organfehlbildungen, geistige Beeinträchtigungen und pädiatrische Tumorerkrankungen/Leukämien 9 10 . Mehrere Studien haben sich mit diesen Risiken bei medizinischem Personal befasst, das am Arbeitsplatz ionisierender Strahlung ausgesetzt ist. Diese Studien haben weitgehend ergeben, dass das fetale Risiko vernachlässigbar ist, wenn angemessene Sicherheitspraktiken eingehalten werden 11 12 13 .

Generell sollten keine Endoskopien durch schwangere Endoskopikerinnen durchgeführt werden, bei denen Röntgenuntersuchungen erforderlich sind. Allerdings ist ein Einsatz von Röntgenstrahlung möglich und auch gesetzlich erlaubt, wenn dies der ausdrückliche Wunsch der schwangeren Endoskopikerin ist und dies auf einer Freiwilligkeit beruht. Die zulässige Strahlenexposition für gebärfähige Frauen und für ungeborene Kinder, die aufgrund der Beschäftigung der Schwangeren exponiert werden, ist in § 78 (4) des Strahlenschutzgesetzes (StrlSchG) festgelegt. Die Organ-Äquivalentdosis des Uterus darf bei gebärfähigen Frauen nicht größer als 2 mSv pro Monat sein, diese Maßnahme dient dem Schutz des Ungeborenen bei unbekannter Schwangerschaft. Bei bekannter Schwangerschaft darf die effektive Dosis des ungeborenen Kindes im Zeitraum zwischen der Bekanntgabe der Schwangerschaft und deren Ende den Wert von 1 mSv nicht überschreiten. Die European Heart Rhythm Association (EHRA) hat 2017 mit Unterstützung der Heart Rhythm Society (HRS) ein detailliertes Konsensus-Dokument über die berufliche Strahlenexposition in der Elektrophysiologie mit speziellem Schwerpunkt auf das weibliche medizinische Personal und die Schwangeren veröffentlicht 14 . Hier werden Risiken der Strahlenexposition, Strategien und Schutzmaßnahmen für den beruflichen Strahlenschutz ausführlich dargelegt und diskutiert.

Der Zutritt schwangerer Frauen zum Kontrollbereich muss vom fachkundigen Strahlenschutzverantwortlichen oder vom Strahlenschutzbeauftragten erlaubt werden (§ 55 Abs. 2 StrlSchV) und dies ist zu dokumentieren. Bei der Anwendung von Röntgenstrahlung in der Endoskopie müssen Überwachungsmaßnahmen sicherstellen, dass der gesetzlich vorgeschriebene besondere Dosisgrenzwert für das ungeborene Kind eingehalten wird (§ 55 Abs. 4 StrlSchV). Bei Schwangeren muss die berufliche Strahlenexposition wöchentlich ermittelt, dokumentiert und mitgeteilt werden (§ 69 Abs. 2 StrlSchV). Verwendet wird hierfür meist, zusätzlich zum amtlichen Personendosimeter, ein (elektronisches) Zweitdosimeter im Rumpfbereich zur arbeitswöchentlichen Ermittlung der Strahlenexposition. So kann die Gebärmutterdosis beziehungsweise die Dosis des ungeborenen Kindes auch unter ungünstigen Expositionsbedingungen sicher abgeschätzt werden***.

Daten des Britischen Instituts für Radiologie zeigen, dass 98,8% aller medizinischen Fachkräfte mit einer beruflichen Strahlenexposition einer jährlichen Strahlendosis von weniger als 1 mSv ausgesetzt sind und nur <0,01% einer Dosis von >5–10 mSv 15 . Nur wenige kleinere Studien haben die spezifische Strahlenexposition des Fötus durch das Tragen eines Dosimeters auf dem Bauch unter einer Röntgenschürze untersucht. Hierbei zeigten sich jeweils keine Expositionswerte über dem Grenzwert von 1 mSv. In einer Studie mit 81 Gefäßchirurginnen und Auszubildenden, die Standard-Schutzkleidung trugen, ergaben die durchschnittlichen Dosimeterwerte eine vernachlässigbare Strahlenbelastung des Fötus 16 . Eine Befragung von 534 schwangeren interventionellen Radiologinnen, von denen die meisten während der Schwangerschaft weiter praktizierten, zeigte keinen Unterschied im Outcome bei der Schwangerschaft und des Fötus im Vergleich zur Allgemeinbevölkerung 11 .

Die allgemeine Forderung, im Kontrollbereich Röntgenschutzkleidung zu tragen (§ 75 Abs. 1a StrlSchV), gilt auch für die schwangere Endoskopikerin. Die DIN EN 61331–3:2016–09 „Strahlenschutz in der medizinischen Röntgendiagnostik – Teil 3: Schutzkleidung, Augenschutz und Abschirmungen für Patienten“ regelt, welche Schutzkleidung notwendig ist. Für die Endoskopie sind eher geschlossene, schwerere Röntgenschutzkleidung zu verwenden (Front 0,35 mm Pb, Rücken 0,25 mm Pb), wobei moderne Mantelschürzen und Zweiteiler (Weste und Rock) eine ideale Passform, guten Tragekomfort und eine deutliche Reduktion der Gewichtsbelastung für die Wirbelsäule bieten und auch für schwangere Ärztinnen gut geeignet sind. Außerdem ist beim Einsatz von Schwangeren die konsequente Umsetzung allgemeiner Strahlenschutzmaßnahmen wichtig, wie Abstand zum Nutzstrahlungsfeld, Nutzung von anlagenbezogenen Strahlungsabschirmungen (Strahlenschutzscheiben, Untertischschutz), Einblendung und eine konsequente Minimierung der Durchleuchtungszeiten 17 .

Im Rahmen der Schwangerschaft kommt es allgemein zu einer verstärkten Belastung des Bewegungsapparates 18 . Durch das zusätzliche Tragen einer Röntgenschürze kann es zu Muskel-Skelett-Beschwerden kommen wie Rückenschmerzen, Nackenschmerzen und Handschmerzen. Zusätzliche prophylaktische Maßnahmen wie Kompressionsstrümpfe, Beckengurt und spezielle Schuhe/Orthesen sind dabei nicht nur in der Endoskopie im Rahmen einer Schwangerschaft sinnvoll.

## Zusammenfassung

Die Endoskopie stellt einen gut planbaren und sicheren Arbeitsplatz für Ärztinnen in der Schwangerschaft und in der Stillzeit dar, wenn bestimmte Grundsätze beachtet werden. Angesichts der steigenden Zahl von Frauen in der Gastroenterologie und der speziellen Herausforderungen während der Schwangerschaft ist es unerlässlich, angepasste Richtlinien und unterstützende Maßnahmen zu entwickeln. Diese sollten sowohl die Gesundheit und Sicherheit der Ärztinnen und des ungeborenen Kindes als auch die Qualität der Patientenversorgung gewährleisten. Durch die Umsetzung dieser Empfehlungen kann die Gastroenterologie als Fachgebiet weiterhin attraktiv und zugänglich für alle Medizinerinnen bleiben, unabhängig von ihrem Lebensabschnitt oder Familienplanungsstatus.

### Anmerkungen

* Geschlechtsneutrale Formulierung: Ausschließlich zum Zweck der besseren Lesbarkeit wird auf die geschlechtsspezifische Schreibweise verzichtet. Alle personenbezogenen Bezeichnungen in diesem Dokument sind somit geschlechtsneutral zu verstehen.

### ^#, †^ Gutachten, Checklisten und weiterführendes Informationsmaterial

Die Deutsche Gesellschaft für Gastroenterologie, Verdauungs- und Stoffwechselkrankheiten (DGVS) stellt ihren Mitgliedern intern das Rechtsgutachten der Medizinkanzlei Mohr „Das ärztliche Arbeiten in der Schwangerschaft im Fachbereich der Gastroenterologie unter spezialisierter Berücksichtigung endoskopischer Untersuchungsleistungen“ auf Anfrage zur Verfügung. Auf der Homepage der DGVS ( https://www.dgvs.de/juga-nachwuchs/schwangerschaft-familie/mit-bauch-und-begeisterung/ ) haben die Autoren und die AG JUGA weiterführende Informationen, Checklisten, Positivlisten, Links und aktuelles Begleitmaterial zusammengestellt.

### Beitrag der Autorinnen und Autoren

Die Beiträge der Autoren sind wie folgt.

Konzeption und Design: Henrike Lenzen und Jutta Keller

Datenerhebung: Henrike Lenzen und Jutta Keller

Entwurf des Manuskripts: Henrike Lenzen und Jutta Keller

Kritische Überarbeitung des Manuskripts hinsichtlich wichtiger intellektueller Inhalte: Lukas Welsch und Stefanie Reichermeier

Review und Freigabe des Artikels: alle Autoren
